# LC-MS-Based Untargeted Metabolic Profiling in Plasma Following Dapagliflozin Administration in Healthy Volunteers

**DOI:** 10.3390/metabo15070484

**Published:** 2025-07-17

**Authors:** Hyeon Ji Kim, Jae Hwa Lee, Ji Seo Park, Jin Ju Park, Hae Won Lee, Heeyoun Bunch, Sook Jin Seong, Mi-Ri Gwon, Young-Ran Yoon

**Affiliations:** 1 Department of Molecular Medicine, School of Medicine, Kyungpook National University, Daegu 41944, Republic of Korea; hyeonjidd@knuh-ext.kr (H.J.K.); leejh0202@knu.ac.kr (J.H.L.); jiseo.park@knu.ac.kr (J.S.P.); pjinju@knu.ac.kr (J.J.P.); haewonbbc@knuh.kr (H.W.L.); wintersj@knu.ac.kr (S.J.S.); 2 Department of Clinical Pharmacology and Therapeutics, Kyungpook National University Hospital, Daegu 41944, Republic of Korea; 3 Department of Applied Biosciences, Kyungpook National University, Daegu 41566, Republic of Korea; hbunch@knu.ac.kr; 4 Clinical Omics Institute, School of Medicine, Kyungpook National University, Daegu 41405, Republic of Korea

**Keywords:** dapagliflozin, healthy individual, untargeted metabolomics, LC-QTOF/MS

## Abstract

Dapagliflozin, a sodium-glucose cotransporter 2 inhibitor, treats type 2 diabetes by blocking renal glucose reabsorption and promoting urinary glucose excretion. This mechanism lowers blood glucose concentrations independently of insulin. The resulting caloric loss also contributes to weight reduction. Although these effects are well documented in patients with diabetes, their magnitude and underlying mechanisms in healthy individuals remain poorly understood. Background/Objectives: We investigated metabolic alterations after a single 10 mg dose of dapagliflozin in healthy adults with normal body-mass indices (BMIs) using untargeted metabolomics. Methods: Thirteen healthy volunteers completed this study. Plasma was collected before and 24 h after dosing. Untargeted metabolic profiling was performed with ultra-high-performance liquid chromatography–quadrupole time-of-flight/mass spectrometry. Results: Twenty-five endogenous metabolites were annotated; ten were putatively identified. Eight metabolites increased significantly, whereas two decreased. Up-regulated metabolites included phosphatidylcholine (PC) species (PC O-36:5, PC 36:3), phosphatidylserine (PS) species (PS 40:2, PS 40:3, PS 36:1, PS 40:4), lysophosphatidylserine 22:1, and uridine. Dehydroepiandrosterone sulfate and bilirubin were down-regulated. According to the Human Metabolome Database, these metabolites participate in glycerophospholipid, branched-chain amino acid, pyrimidine, and steroid-hormone metabolism. Conclusions: Dapagliflozin may affect pathways related to energy metabolism and homeostasis beyond glucose regulation. These data provide a reference for future investigations into energy balance and metabolic flexibility in metabolic disorders.

## 1. Introduction

Dapagliflozin is a widely prescribed medication for treating type 2 diabetes (T2D). It belongs to the sodium–glucose cotransporter 2 (SGLT2) inhibitor class, commonly known as gliflozins [1]. By inhibiting SGLT2 proteins in the proximal renal tubules, dapagliflozin promotes urinary glucose excretion and thereby lowers blood glucose levels [2]. The resultant osmotic diuresis [3] produces caloric loss, weight reduction, and decreased fat mass [4]. The mechanism by which dapagliflozin induces weight loss differs from that of other antihyperglycaemic agents, such as biguanides and glucagon-like peptide-1 (GLP-1) receptor agonists. Biguanides reduce hepatic glucose production through activation of 5′ adenosine monophosphate-activated protein kinase, thereby lowering blood glucose concentrations [5]. GLP-1 receptor agonists suppress appetite, stimulate glucose-dependent insulin secretion, and inhibit glucagon release [6]. In contrast, dapagliflozin does not directly influence appetite or insulin sensitivity [7].

Stable energy metabolism is essential for long-term health, and cardiovascular and metabolic diseases remain major contributors to global disease burden [8]. Given the widespread use of SGLT2 inhibitors, understanding their pharmacometabolic effects in healthy individuals may clarify how these agents modulate energy metabolism independent of underlying disease [2,4,9]. Notably, the magnitude of these effects may vary depending on an individual’s baseline metabolic state, including insulin sensitivity and body composition [10].

A comprehensive analytical approach—specifically, metabolomics—is therefore required to investigate dapagliflozin-induced metabolic shifts. Targeted metabolomics focuses on predefined metabolites or pathways and may overlook unexpected drug-related alterations [11]. By contrast, untargeted metabolomics captures a broad spectrum of metabolites without prior knowledge of the metabolome, offering a holistic view of metabolic adaptations after drug administration [1]. This strategy is particularly effective for exploring complex biological systems and identifying potential biomarkers [12], thereby providing deeper insight into drug-mediated perturbations of energy metabolism and related pathways. For example, Bletsa et al. used untargeted metabolomics to examine urinary metabolite changes in patients with T2D after dapagliflozin therapy [13]. Their study revealed pronounced shifts in energy metabolism and renal excretion—such as elevated branched-chain amino acids like leucine—alongside markers of fatty acid oxidation and ketogenesis [1,13]. These findings underscore the capacity of untargeted profiling to uncover multifaceted metabolic changes that extend beyond glucose regulation.

Although extensive research has documented the effects of dapagliflozin in individuals with diabetes [2,4], its metabolic impact on people without diabetes—including healthy populations—remains insufficiently explored. Addressing this knowledge gap is essential for achieving a comprehensive understanding of how dapagliflozin modulates metabolic pathways across diverse population groups. Accordingly, the present study investigated endogenous metabolic changes induced by dapagliflozin in healthy, normal-weight individuals using an untargeted metabolomics approach.

## 2. Materials and Methods

### 2.1. Study Design and Sample Collection

This study protocol was approved by the Institutional Review Board at Kyungpook National University Hospital, Daegu, Republic of Korea (KNUH 2022-09-016-001). This study was registered with the Clinical Research Information Service (KCT0007864), and written informed consent was obtained from all participants. Healthy volunteers were enrolled according to predefined inclusion and exclusion criteria: age ≥ 19 years, body mass index (BMI) 18.0–25.0 kg/m^2^, and no evidence of congenital or chronic disease on physical examination, clinical laboratory testing, or electrocardiography. Exclusion criteria included a history of diabetes mellitus; clinically significant hepatic, renal, or endocrine disorders; abnormal liver function (aspartate aminotransferase, alanine aminotransferase, or total bilirubin > 1.5 × upper normal limit); impaired renal function (creatinine clearance < 80 mL/min); or inherited disorders of carbohydrate metabolism such as galactose intolerance, Lapp lactase deficiency, or glucose–galactose malabsorption. A single oral dose of dapagliflozin (Forxiga^®^, AstraZeneca, Cambridge, UK) 10 mg was administered, and blood samples for baseline clinical characteristics were collected before dosing. Plasma samples for metabolic profiling were obtained immediately before (pre) and 24 h after (post) administration. Whole blood was transferred to heparinized tubes, centrifuged at 1900× *g* for 10 min at 4 °C, and the plasma was stored at −80 °C until analysis.

### 2.2. Chemicals and Reagents

High-performance liquid chromatography (HPLC)-grade methanol and acetonitrile were purchased from Merck (Darmstadt, Germany). Formic acid (liquid chromatography–mass spectrometry (LC-MS) grade, >98.0%) and acetic acid (LC-MS grade, >99.5%) were obtained from Tokyo Chemical Industry Co., Ltd. (Tokyo, Japan). Deionized water was produced with a Milli-Q^®^ Direct 16 Water Purification System (Millipore SAS, Guyancourt, France). Hexakis (2,2-difluoroethoxy)phosphazene, used as an external standard, was supplied by Apollo Scientific Ltd. (Bredbury, UK). A 10 mM sodium formate solution was prepared by diluting 10 M sodium hydroxide (Sigma-Aldrich, St. Louis, MO, USA) with deionized water and 2-propanol (Thermo Fisher Scientific, Waltham, MA, USA) in a 1:1 (*v/v*) ratio, followed by the addition of 0.2% formic acid. This solution was applied prior to batch acquisition to generate a series of cluster ions that served as reference peaks for adjusting the mass axis and ensuring mass accuracy across the full scan range.

### 2.3. Untargeted Metabolic Profiling

#### 2.3.1. Sample Preparation

Plasma samples were thawed at 4 °C. Equal aliquots from each sample were pooled to generate quality-control (QC) material. To monitor system stability, pooled QC samples were analyzed intermittently throughout the batch.

For protein precipitation, 150 µL cold methanol was added to 50 µL plasma and vortex-mixed for 10 min. After centrifugation at 21,300× *g* for 15 min at 4 °C, 100 µL supernatant was transferred to a 2.0 mL polypropylene tube and evaporated under vacuum at 45 °C for 2 h. The residue was reconstituted in 200 µL methanol, vortexed for 10 min, and recentrifuged (21,300× *g*, 4 °C). The final supernatant was placed in autosampler vials for analysis.

#### 2.3.2. Liquid Chromatography–Tandem MS Conditions

Untargeted metabolic profiling was performed on an ultra-high-performance liquid chromatography–quadrupole time-of-flight MS (UHPLC-QTOF/MS) platform. Separation was achieved on a Thermo Scientific Dionex UltiMate 3000 UHPLC system (Dionex Softron GmbH, Germering, Germany) equipped with a Waters ACQUITY UPLC^®^ BEH C18 column (100 × 2.1 mm, 1.7 µm, 130 Å; Waters, Milford, MA, USA). The column was maintained at 50 °C and the autosampler at 4 °C. Injection volume was 2 µL, and the flow rate was 0.2 mL/min.

For positive ionization mode, the mobile phase consisted of 0.1% formic acid in deionized water (A) and 0.1% formic acid in methanol (B). The gradient was as follows: 0.0–0.5 min, 1% B; 0.5–1.0 min, 65% B; 1.0–4.0 min, 90% B; 4.0–12.0 min, 99% B; 12.0–22.0 min, held at 99% B; 22.0–22.5 min, 1% B; and 22.5–26.5 min, 1% B.

For negative ionization mode, the mobile phases comprised 0.02% acetic acid in deionized water (A) and 0.02% acetic acid in acetonitrile (B). The gradient was as follows: 0.0–0.5 min, 1% B; 0.5–1.0 min, 65% B; 1.0–4.0 min, 90% B; 4.0–19.0 min, 90% B; 19.0–19.5 min, 99% B; 19.5–22.5 min, held at 99% B; 22.5–23.0 min, 1% B; and 23.0–28.5 min, 1% B.

MS analysis was performed on a Bruker Compact^™^ mass spectrometer (Bruker Daltonics GmbH & Co., KG, Bremen, Germany) equipped with an electrospray ionization source. The MS parameters were set as follows: mass scan range, 50–1000 *m*/*z*; nebulizer gas pressure, 0.8 bar; dry gas flow rate, 10.0 L min^−1^; dry gas temperature, 200 °C; capillary voltage, ±4500 V; and end-plate offset, −500 V.

Tandem MS (MS/MS) analysis was carried out to obtain fragmentation patterns of metabolites. Nitrogen served as the collision gas. MS/MS spectra were acquired over a scan range of 50–1000 *m*/*z*. Accurate-mass data were collected at 4 spectra s^−1^, with the five most abundant precursor ions selected for MS/MS scans. To prevent fragmentation of the external standard at [M + H]^+^ 622.0289 *m*/*z*, the 620–625 *m*/*z* range was excluded in positive ionization mode. In negative ionization mode, the ranges 555–558 *m*/*z* (for [M−C2F2H3]^−^ 556.0020 *m*/*z*) and 680–684 *m*/*z* (for [M + CH3COOH−H]^−^ 680.0344 *m*/*z*) were similarly excluded. Precursor ions released after 30 s were omitted from further analysis. Fragmentation of precursor ions at 100, 300, 500, and 1000 *m*/*z* in positive ionization mode was achieved with collision energies of 5, 12, 20, and 50 eV, respectively. In negative ionization mode, precursor ions at 100, 300, 500, 700, and 1000 *m*/*z* were fragmented with collision energies of 5, 10, 16, 20, and 30 eV, respectively.

### 2.4. Data Analysis

Raw spectrometric data were converted into a feature table using MetaboScape^®^ version 5.0 (Bruker Daltonics GmbH & Co., KG, Bremen, Germany). Data normalization, including quantile normalization and log transformation, was performed using R version 4.3.3 (https://www.r-project.org, accessed on 29 March 2024) on raw peak intensity values. Periodically injected pooled QC samples were included to monitor instrument stability and reduce technical variation across samples. SIMCA version 17.0 (Sartorius Stedim Data Analytics AB, Umeå, Sweden) was used for Pareto scaling and multivariate statistical analysis. Class clustering was evaluated with principal component analysis and orthogonal partial least squares–discriminant analysis (OPLS-DA). The OPLS-DA model was validated by 100-iteration permutation testing. Model quality was assessed using R^2^Y (goodness of fit) and Q^2^ (predictive ability). Variable importance in projection (VIP) values were calculated from the OPLS-DA models to determine the contribution of each metabolic feature to group discrimination.

### 2.5. Metabolite Annotation

Metabolic features with a VIP value ≥ 1.0 were selected for annotation. Within MetaboScape^®^ version 5.0, features exhibiting mass accuracy within ±2–5 ppm of the theoretical *m*/*z* were retained to ensure reliable elemental composition assignments. Isotopic pattern quality was evaluated using mSigma values within a tolerance range of 50–100, ensuring that the observed isotopic distribution closely matched the theoretical pattern derived from the elemental formula. For MS/MS-based annotation, acquired fragmentation spectra were compared against reference spectra available in both internal libraries embedded within MetaboScape and publicly accessible online databases, including the Chemical Entities of Biological Interest (ChEBI, https://www.ebi.ac.uk/chebi/init.do, accessed on 7 May 2024), Human Metabolome Database (HMDB, https://hmdb.ca, accessed on 7 May 2024), LIPID MAPS (https://www.lipidmaps.org, accessed on 7 May 2024), MS-DIAL version 4.9 (https://systemsomicslab.github.io/compms/msdial/main.html, accessed on 24 May 2024), and PubChem (https://pubchem.ncbi.nlm.nih.gov, accessed on 7 May 2024).

### 2.6. Statistical Analysis

All statistical analyses were performed in JASP version 0.18.3.0 (https://jasp-stats.org, accessed on 15 April 2024). For normally distributed data, *p*-values were calculated with a paired-sample *t*-test; otherwise, the Wilcoxon signed-rank test was applied. False discovery rate (FDR)-adjusted *p*-values were calculated using the Benjamini–Hochberg method, with a *q*-value < 0.05 considered statistically significant.

## 3. Results

### 3.1. Demographic and Clinical Characteristics

Thirteen of the fifteen enrolled participants completed this study. Table 1 summarizes their demographic and clinical characteristics before dapagliflozin administration. All parameters lay within normal reference ranges, confirming that the cohort was clinically healthy.

### 3.2. Metabolic Profiling and Multivariate Analysis

**Figure 1:**
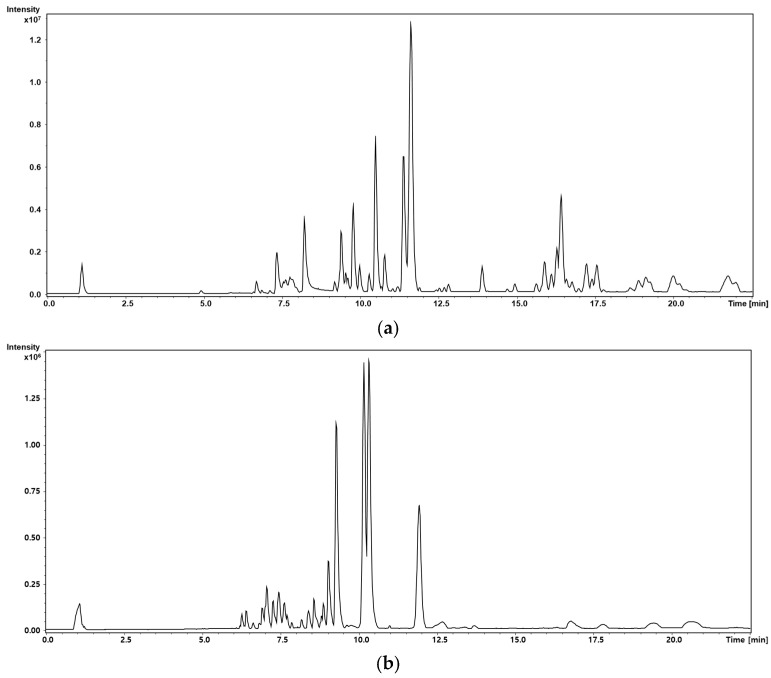
Representative base-peak chromatograms of pooled quality-control samples in (**a**) positive and (**b**) negative ionization modes.

To explore endogenous metabolic alterations induced by dapagliflozin, untargeted metabolic profiling was performed on plasma samples using UHPLC-QTOF/MS. Figure 1 presents representative base-peak chromatograms of pooled QC samples acquired in (a) positive and (b) negative ionization modes, illustrating clear peak separation.

**Figure 2:**
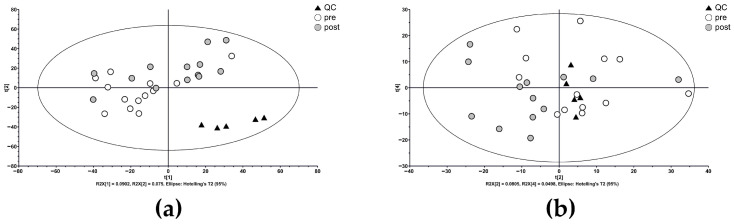
Principal component analysis score plots of metabolic profiles in (**a**) positive and (**b**) negative ionization modes. The plots demonstrate clustering of quality-control samples and subtle time-point separation, suggesting potential dapagliflozin-induced metabolic changes.

Principal component analysis score plots (Figure 2) confirmed tight clustering of QC samples, with relative standard deviations of 5.3% and 6.8% in the (a) positive and (b) negative modes, respectively, indicating consistent instrument performance. As expected, no obvious separation between pre- and post-administration samples was observed in the unsupervised model.

**Figure 3:**
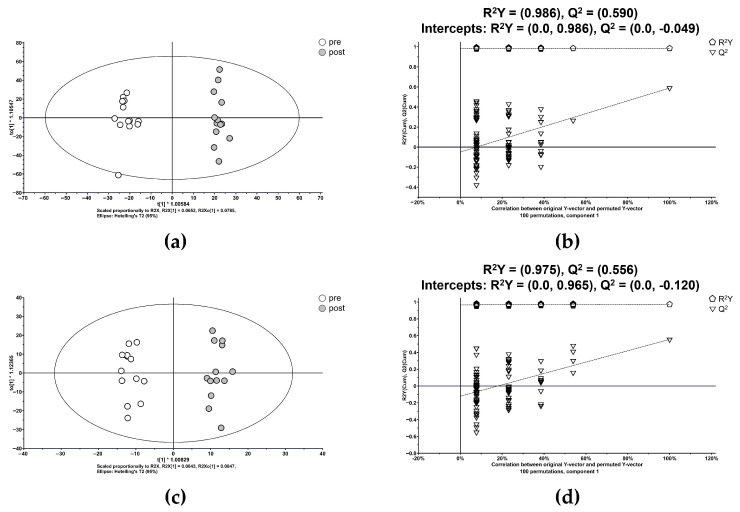
Orthogonal partial least squares discriminant analysis score plots and permutation tests comparing samples collected before and after a single dose of dapagliflozin. Panels (**a**,**b**) depict the positive-mode score and permutation plots, while (**c**,**d**) show the negative-mode equivalents.

Supervised OPLS-DA highlighted significant metabolic alterations, yielding R^2^Y = 0.986 and Q^2^ = 0.590 in the (a) positive mode and R^2^Y = 0.975 and Q^2^ = 0.556 in the (b) negative mode (Figure 3). Permutation tests confirmed model robustness and the metabolic distinction between pre- and post-administration states.

### 3.3. Metabolite Annotation

Metabolite features with VIP ≥ 1.0 were annotated. Features that additionally met the FDR threshold of *q* < 0.05 were considered putative metabolites. In total, 25 endogenous metabolites were assigned, including 10 putative compounds (Table 2). In the positive ion mode, the putative metabolites comprised phosphatidylcholine (PC) species—PC O-36:5 and PC 36:3—as well as uridine and bilirubin. In the negative ion mode, the annotated metabolites included phosphatidylserine (PS) species—PS 40:2, PS 40:3, PS 36:1, and PS 40:4—lysophosphatidylserine (Lyso-PS) 22:1, and dehydroepiandrosterone sulfate (DHEA-S).

Following dapagliflozin administration, significant increases were observed in PC O-36:5, PC 36:3, PS 40:2, PS 40:3, PS 36:1, Lyso-PS 22:1, PS 40:4, and uridine. PC—including the variants PC O-36:5 and 36:3—belongs to the glycerophospholipid class and is a major constituent of cellular membranes, as well as a participant in lipid metabolism and cell signaling. Likewise, PS—which includes PS 40:2, 40:3, 36:1, and 40:4—also resides in cellular membranes and contributes to membrane structure, apoptosis, and signaling. Lyso-PS 22:1, a lysophospholipid derived from PS hydrolysis, supports membrane remodeling and plays key roles in inflammatory responses and signaling. Leucine, one of the three branched-chain amino acids, contributes to protein synthesis signaling and energy metabolism. Uridine, a nucleoside, is central to pyrimidine metabolism, nucleotide synthesis, and systemic energy regulation.

Conversely, DHEA-S and bilirubin levels declined after dapagliflozin exposure. DHEA-S, a sulfated steroid, mediates adaptive responses to reduced glucose availability and shifts in energy metabolism, whereas bilirubin acts as an antioxidant that protects cells from oxidative damage.

## 4. Discussion

This study focused on the acute metabolic changes induced by dapagliflozin administration in metabolically healthy individuals. This untargeted metabolomics analysis was pre-specified to include only participants within a restricted BMI range (18.0–25.0), aiming to evaluate drug-induced metabolic responses in a metabolically stable population. This design reduced potential confounding related to baseline metabolic heterogeneity. Untargeted metabolic profiling with UHPLC-QTOF/MS revealed observable alterations after a single dose of dapagliflozin: phospholipids, leucine, and uridine increased, whereas DHEA-S and bilirubin decreased.

**Figure 4:**
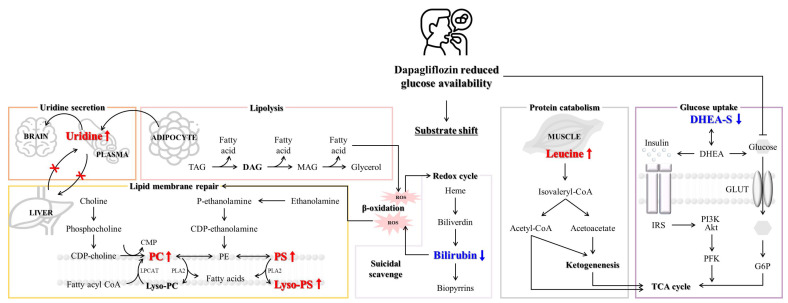
Systematic diagram illustrating metabolic adaptations after dapagliflozin administration. Dapagliflozin elicits systemic metabolic changes that conserve energy and counteract oxidative stress. Elevated uridine attenuates thermogenesis and lowers metabolic rate. Increased phospholipids reflect membrane repair in response to ROS generated during β-oxidation. Leucine, derived from muscle catabolism, supports ketogenesis and energy production. Reduced DHEA-S suggests enhanced glucose uptake via conversion to DHEA, whereas bilirubin depletion underscores its antioxidant role in neutralizing oxidative stress. Red text with upward arrows (↑) indicates increased metabolite levels; blue text with downward arrows (↓) indicates decreased levels. The × symbol denotes suppressed liver–plasma uridine exchange. TAG, triacylglycerol; DAG, diacylglycerol; MAG, monoacylglycerol; CDP, cytidine diphosphate; CoA, coenzyme A; CMP, cytidine monophosphate; PC, phosphatidylcholine; PE, phosphatidylethanolamine; PS, phosphatidylserine; LPCAT, lysophosphatidylcholine acyltransferase; PLA2, phospholipase A2; Lyso-PC, lysophosphatidylcholine; Lyso-PS, lysophosphatidylserine; ROS, reactive oxygen species; DHEA-S, dehydroepiandrosterone sulfate; DHEA, dehydroepiandrosterone; GLUT, glucose transporter; G6P, glucose-6-phosphate; PFK, phosphofructokinase; Akt, protein kinase B; PI3K, phosphoinositide 3-kinase; IRS, insulin receptor substrate; TCA cycle, tricarboxylic acid cycle.

These results demonstrate that the administration of dapagliflozin mimics the early metabolic response to glucose deprivation. This observation aligns with the well-established human adaptation to fasting, during which lipids supplant carbohydrates as the primary energy source once glycogen stores are depleted [14]. In this state, fatty acids undergo β-oxidation to generate ketone bodies, which serve as an alternative fuel source [15]. Figure 4 illustrates that the metabolic profile induced by dapagliflozin mirrors the adaptation to glucose deprivation; underscoring the drug’s influence on energy homeostasis.

Under caloric restriction or glucose depletion, adipocytes initiate lipolysis, hydrolyzing triacylglycerol into diacylglycerol, monoacylglycerol, fatty acids, and glycerol to mobilize fatty acids for energy [16]. The liberated fatty acids travel to the liver, where they undergo β-oxidation in mitochondria [17]. This pathway generates acetyl coenzyme A, reduced flavin adenine dinucleotide, and reduced nicotinamide adenine dinucleotide, which feed the electron transport chain to produce adenosine triphosphate (ATP) [18].

However, elevated β-oxidation increases electron leakage from electron transport chain complexes, forming superoxide radicals [19]. These radicals serve as precursors to reactive oxygen species (ROS), inducing oxidative stress [19]. Excess ROS oxidizes amino-acid residues, impairs enzyme activity, and disrupts protein folding [20]. In parallel, ROS-driven lipid peroxidation yields malondialdehyde and 4-hydroxynonenal, further propagating oxidative stress and exacerbating mitochondrial dysfunction [21]. Such peroxidation compromises the phospholipid bilayer, increasing membrane permeability and reducing fluidity [22,23]. Enhanced β-oxidation during glucose scarcity, therefore, amplifies oxidative damage [23]. To counteract this stress, cells are known to activate membrane-repair or remodeling mechanisms [24], including up-regulation of phospholipid synthesis to maintain membrane integrity [25]. Diacylglycerol generated from triacylglycerol hydrolysis feeds into the synthesis of PC, phosphatidylethanolamine, and PS [26]. PC and PS can then be hydrolyzed by phospholipase A2 to yield lysophosphatidylcholine (Lyso-PC) and Lyso-PS [27]; Lyso-PC may also be reacylated to PC via Lyso-PC acyltransferase [28]. The observed increases in PC, PS, and Lyso-PS after dapagliflozin may reflect membrane or cellular adaptation to metabolic stress due to glucose loss. According to Pamplona [29], phospholipid remodeling can occur in response to oxidative or energetic stress, which may explain these changes. Measuring markers of oxidative stress or lipid oxidation would help elucidate the underlying mechanisms and further support the biological interpretation of these phospholipid alterations.

Acting as a suicidal scavenger, bilirubin mitigates ROS-induced damage [30]. In a redox cycle, bilirubin neutralizes ROS by cycling with its oxidized form, biliverdin [31]. This cycle significantly reduces oxidative stress and safeguards cellular membranes, proteins, and deoxyribonucleic acid from ROS-induced damage [32]. In this study, circulating bilirubin levels decreased following dapagliflozin administration. Although fasting and energy depletion are typically associated with increased bilirubin as a protective response [33], the observed decrease may suggest either enhanced bilirubin consumption in response to transient oxidative stress or a reduced demand for antioxidant buffering due to metabolic reprogramming and attenuated ROS production. The rise in leucine observed in this study may reflect up-regulated protein catabolism in muscle tissue, a metabolic adaptation to reduced glucose availability [34]. While leucine did not reach statistical significance after multiple testing correction, its known involvement in energy metabolism provides a useful context for interpreting the observed metabolic patterns. As a ketogenic amino acid, leucine is metabolized to acetyl coenzyme A and acetoacetate [35]. Acetyl coenzyme A enters the tricarboxylic acid cycle to support energy production or fuel ketogenesis, generating ketone bodies such as acetoacetate [36]. These ketone bodies serve as alternative energy substrates, primarily for the brain and liver, thereby sustaining ATP production [15]. This observation aligns with previous reports that skeletal-muscle protein metabolism is highly sensitive to energy deprivation [37]. During glucose scarcity, the body likely shifts to protein-derived substrates to satisfy energy demands, a compensatory response that highlights the interplay among protein catabolism, amino acid metabolism, and ketogenesis in maintaining energy homeostasis [37,38]. While ketone bodies were not directly measured, the observed increase in leucine may suggest engagement of ketogenesis-related pathways, which have been reported under conditions of glucose scarcity. Additional measurement of ketone bodies would allow more definitive mechanistic insight.

DHEA-S, a steroid hormone, undergoes enzymatic conversion to its bioactive form, DHEA, via tissue-specific activity of steroid sulfatases [39]. DHEA helps meet metabolic demands by activating the phosphoinositide 3-kinase/Akt pathway in skeletal muscle and adipose tissue [40]. Activation of this pathway facilitates glucose-transporter translocation to the plasma membrane, enhancing glucose uptake and potentially improving insulin sensitivity [40,41]. During glucose scarcity, the decrease in circulating DHEA-S observed in this study may reflect increased desulfation to DHEA in peripheral tissues. This possibility is supported by Marciniak et al. [42], who reported elevated DHEA levels following short-term fasting, suggesting a compensatory response to metabolic stress.

Uridine is synthesized in adipocytes during fasting via the de novo pyrimidine-biosynthesis pathway and released into the bloodstream to regulate systemic energy balance [43]. During fasting, hepatic involvement in uridine regulation wanes, and plasma levels become increasingly dependent on adipocyte-derived uridine [44]. Elevated plasma uridine acts on the hypothalamus, enhancing thermoregulation that lowers body temperature and metabolic rate, thereby reducing energy expenditure [43]. Following dapagliflozin administration, reduced glucose availability mimics a fasting state; the increase in uridine detected in this study, therefore, suggests a metabolic adaptation linked to SGLT2-inhibition-induced alterations in glucose homeostasis.

This study has several limitations. First, the metabolites annotated by untargeted metabolomics are classified as putative level-2 metabolites according to the Metabolomics Standards Initiative [45]. Second, as dapagliflozin is administered once daily, the 24 h post-dose sample was intentionally selected to reflect integrated metabolic response over a full dosing interval. Nonetheless, the small sample size and single-dose design limit the generalizability of our findings to chronic or time-dependent metabolic effects.

## 5. Conclusions

In this study, we examined the acute metabolic effects of dapagliflozin in healthy individuals using untargeted metabolomics. Dapagliflozin-induced urinary glucose excretion was accompanied by metabolic adaptations that may reflect early shifts toward enhanced fatty acid oxidation, protein catabolism, and the engagement of alternative energy pathways. These adaptations were potentially accompanied by mechanisms that preserve cellular integrity and mitigate oxidative stress, underscoring the complex interplay between energy regulation and metabolic flexibility under reduced glucose availability. Overall, our findings highlight the influence of dapagliflozin on lipid and amino acid metabolism beyond glucose regulation, even in non-diabetic populations. The observed metabolic shifts deepen our understanding of energy regulation and lay the groundwork for future research into metabolic disorders such as obesity and diabetes. Moreover, they provide a rationale for long-term studies exploring the effects of dapagliflozin and other SGLT2 inhibitors across diverse populations.

## Figures and Tables

**Table 1:** Demographic and clinical characteristics of the 13 participants prior to dapagliflozin administration.

Variable	Mean ± Standard Deviation
Age (years)	29.6 ± 8.9
Height (cm)	174.9 ± 6.3
Weight (kg)	70.4 ± 8.7
BMI (kg/m^2^)	22.9 ± 1.9
WBC (10^3^/mL)	6.2 ± 1.7
Hemoglobin (g/mL)	15.4 ± 0.7
Hematocrit (%)	46.1 ± 2.0
Platelets (10^3^/mL)	266.8 ± 44.7
Total protein (g/dL)	7.6 ± 0.4
Albumin (g/dL)	4.9 ± 0.2
Total bilirubin (mg/dL)	0.8 ± 0.2
AST (U/L)	20.1 ± 5.4
ALT (U/L)	19.6 ± 9.1
Alkaline phosphatase (U/L)	79.7 ± 20.9
Fasting glucose (mg/dL)	97.5 ± 6.7
BUN (mg/dL)	12.5 ± 2.8
Creatinine (mg/dL)	0.9 ± 0.1
Total cholesterol (mg/dL)	181.2 ± 23.1

Abbreviations: BMI, body mass index; WBC, white blood cell count; AST, aspartate transaminase; ALT, alanine transaminase; BUN, blood urea nitrogen.

**Table 2:** Metabolic alterations following dapagliflozin administration.

Metabolite	VIP	RT (min)	*m*/*z*	Ion Type	Database	FC ^§^	FDR ^¶^
Fatty acyls							
13-Docosenamide	2.27	12.65	360.3250	[M + H]^+^	HMDB0244507	0.965	0.153
NAGlySer 31:1	2.10	17.68	639.4971	[M + H]^+^	Pubchem:164316244	0.974	0.219
Octadecanoic acid	1.78	14.62	355.2840	[M − H]^−^	HMDB0000827	0.939	0.128
5-Hexyltetrahydro-2-furanoctanoic acid	1.46	8.27	297.2418	[M − H]^−^	HMDB0031127	0.978	0.280
Glycerolipids							
DG 40:6	2.04	19.55	669.5437	[M + H]^+^	ChEBI:85709	0.976	0.260
DG 38:4	1.37	20.25	645.5444	[M + H]^+^	ChEBI:85705	0.985	0.247
Phospholipids							
PC O-36:5	2.73	16.29	766.5741	[M + H]^+^	HMDB0013415	1.066	0.038
PC 36:4	2.22	15.80	782.5730	[M + H]^+^	ChEBI:64524	1.031	0.056
PC 36:3	1.87	16.58	784.5912	[M + H]^+^	ChEBI:64523	1.021	0.019
SM 51:9;2O	1.84	21.74	925.7112	[M + H]^+^	PubChem:164450933	1.064	1.000
PS 40:2	1.73	21.07	842.5909	[M − H]^−^	ChEBI:141307	1.030	0.008
PS 40:3	1.72	16.99	840.5778	[M − H]^−^	ChEBI:141308	1.035	0.032
PC 36:5	1.58	15.62	780.5553	[M + H]^+^	ChEBI:64504	1.022	0.057
PS 36:1	1.52	16.09	788.5470	[M − H]^−^	ChEBI:136256	1.039	0.048
Lyso-PS 22:1	1.46	7.07	578.3479	[M − H]^−^	ChEBI:72415	1.021	0.080
Lyso-PS 22:1	1.42	6.92	578.3474	[M − H]^−^	ChEBI:72415	1.019	0.022
PS 40:4	1.35	16.35	838.5631	[M − H]^−^	PubChem:138143401	1.033	0.024
PC 42:9	1.33	17.18	856.5841	[M + H]^+^	ChEBI:136099	0.996	0.295
Lyso-PC 22:6	1.32	9.26	568.3409	[M + H]^+^	HMDB0010404	0.996	0.402
PC 34:3	1.13	15.49	756.5535	[M + H]^+^	ChEBI:64424	1.005	0.474
PS 42:2	1.03	11.16	870.6242	[M − H]^−^	PubChem:138212963	1.018	0.423
PS 38:0	1.01	8.30	818.5938	[M − H]^−^	PubChem:75958984	1.019	0.295
Sterol lipids							
DHEA-S	2.54	12.33	367.1563	[M − H]^−^	HMDB0001032	0.924	0.033
Amino acids and nucleotides							
Leucine	1.44	1.98	132.1021	[M + H]^+^	HMDB0000687	1.024	0.051
Uridine	1.20	6.61	245.0788	[M + H]^+^	HMDB0000296	1.008	0.020
Bile pigments							
Bilirubin	4.11	6.10	585.2711	[M + H]^+^	HMDB0000054	0.910	0.003

^§^ Fold change was calculated as the ratio of post- to pre-administration levels. ^¶^ False discovery rate was computed using the Benjamini–Hochberg method for multiple testing correction. Abbreviations: VIP, variable importance in projection; RT, retention time; *m*/*z*, mass-to-charge ratio; FC, fold change; FDR, false discovery rate; NAGlySer, N-acyl glycyl serine; DG, diacylglycerol; PC, phosphatidylcholine; SM, sphingomyelin; PS, phosphatidylserine; Lyso-PS, lysophosphatidylserine; Lyso-PC, lysophosphatidylcholine; DHEA-S, dehydroepiandrosterone sulfate; HMDB, Human Metabolome Database; ChEBI, Chemical Entities of Biological Interest; PubChem, Public Chemical Database (U.S. National Institutes of Health).

## Data Availability

Raw data are available at the Korea BioData Station (K-BDS, https://kbds.re.kr/ (accessed on 22 November 2024)) under accession ID KAP241008.

## Abbreviations

Akt	Protein kinase B
ALT	Alanine Transaminase
AST	Aspartate Transaminase
ATP	Adenosine Triphosphate
BMI	Body Mass Index
BUN	Blood Urea Nitrogen
CDP	Cytidine Diphosphate
ChEBI	Chemical Entities of Biological Interest
CMP	Cytidine Monophosphate
CoA	Coenzyme A
DAG	Diacylglycerol
DG	Diacylglycerol
DHEA	Dehydroepiandrosterone
DHEA-S	Dehydroepiandrosterone Sulfate
FC	Fold Change
FDR	False Discovery Rate
G6P	Glucose-6-Phosphate
GLP-1	Glucagon-Like Peptide-1
GLUT	Glucose Transporter
HMDB	Human Metabolome Database
HPLC	High-Performance Liquid Chromatography
IRS	Insulin Receptor Substrate
LC-MS	Liquid Chromatography–Mass Spectrometry
LPCAT	Lysophosphatidylcholine Acyltransferase
Lyso-PC	Lysophosphatidylcholine
Lyso-PS	Lysophosphatidylserine
*m*/*z*	Mass-to-charge ratio
MAG	Monoacylglycerol
MS	Mass Spectrometry
MS/MS	Tandem Mass Spectrometry
NAGlySer	N-acyl glycyl serine
OPLS-DA	Orthogonal Partial Least Squares–Discriminant Analysis
PC	Phosphatidylcholine
PE	Phosphatidylethanolamine
PFK	Phosphofructokinase
PI3K	Phosphoinositide 3-Kinase
PLA2	Phospholipase A2
PS	Phosphatidylserine
QC	Quality Control
ROS	Reactive Oxygen Species
RT	Retention Time
SGLT2	Sodium–Glucose Cotransporter 2
SM	Sphingomyelin
T2D	Type 2 Diabetes
TAG	Triacylglycerol
TCA cycle	Tricarboxylic Acid Cycle
UHPLC-QTOF/MS	Ultra-High-Performance Liquid Chromatography–quadrupole time-of-flight MS
VIP	Variable Importance in Projection
WBC	White Blood Cell Count
